# Do Volunteers Make a Difference on Nursing Home Quality Measures?

**DOI:** 10.1093/geroni/igaa057.1229

**Published:** 2020-12-16

**Authors:** Paul Falkowski, Christopher Kelly, Nancy Kelley

**Affiliations:** 1 University of Nebraska - Omaha, Philadelphia, Pennsylvania, United States; 2 University of Nebraska Omaha, Omaha, Nebraska, United States; 3 University of Nebraska - Omaha, Omaha, Nebraska, United States

## Abstract

The purpose of this study was to explore the relationship between nursing home volunteer programming and quality measures and increase the knowledge base of nursing home volunteer programming in various settings. Fifty-two nursing homes were surveyed using electronic surveys and personal interviews. Questions focused on the organization of the nursing home, characteristics of the volunteer program and volunteer activities. Of the 52 facilities surveyed, 19 were not part of a chain, 37 were in urban settings, and 24 were for-profit entities. Volunteers were used in 46 nursing homes with a mean number of volunteers of 51.7 onsite an average of 4.9 days per week. Bivariate analysis revealed statistically significant correlations (p<.05) between organizational and volunteer programming characteristics and six quality measures (pressure sores, urinary tract infections, depression, use of restraints, falls, use of antipsychotic and hypnotic drugs). Statistically significant (p<.05) inverse relationships were found between volunteers providing individualized activities (e.g., feeding assistance, combing hair, doing nails, and letter writing) and the incidence of urinary tract infections and the use of psychotropic drugs. Multiple regression analysis revealed a statistically significant (p<.05) inverse relationship between personal volunteer services such as combing hair and doing nails and the use of hypnotic drugs and antipsychotic drugs. This study indicates a significant correlation between volunteer programming and quality measure scores. A larger study of these relationships is indicated.

